# A Few *Pseudomonas* Oligotypes Dominate in the Meat and Dairy Processing Environment

**DOI:** 10.3389/fmicb.2017.00264

**Published:** 2017-03-02

**Authors:** Giuseppina Stellato, Daniel R. Utter, Andy Voorhis, Maria De Angelis, A. Murat Eren, Danilo Ercolini

**Affiliations:** ^1^Division of Microbiology, Department of Agricultural Sciences, University of Naples Federico IIPortici, Italy; ^2^Department of Organismic and Evolutionary Biology, Harvard University, CambridgeMA, USA; ^3^Josephine Bay Paul Center for Comparative Molecular Biology and Evolution, Marine Biological Laboratory, Woods HoleMA, USA; ^4^Department of Soil, Plant and Food Science, University of Bari Aldo MoroBari, Italy; ^5^Department of Medicine, University of Chicago, ChicagoIL, USA

**Keywords:** *Pseudomonas fragi*, food contamination, food processing environment, oligotyping, 16S rRNA gene sequencing

## Abstract

The occurrence of bacteria in the food processing environments plays a key role in food contamination and development of spoilage. Species of the genus *Pseudomonas* are recognized as major food spoilers and the capability to actually determine spoilage can be species- as well as strain-dependent. In order to improve the taxonomic resolution of 16S rRNA gene amplicons, in this study we used oligotyping to investigate the diversity of *Pseudomonas* populations in meat and dairy processing environments. Sequences of the V1–V3 regions from previous studies were used, including environmental swabs and food samples from both meat and dairy processing plants. We showed that the most frequently found oligotypes belonged to *Pseudomonas fragi* and *P. fluorescens*, that the most abundant oligotypes co-occurred, and were shared between the meat and dairy datasets. All the oligotypes occurring in foods were also identified in the environmental samples of the corresponding plants, highlighting the important role of the environment as a source of strains for food contamination. Oligotypes of the same species showed different levels depending on food processing and type of sample, suggesting that different strains of the same species can have different adaptation efficiency, leading to resilient bacterial associations.

## Introduction

The processing environment can be a fundamental source of food contamination across the food chains. This is particularly challenging especially for fresh foods or for those types of products that are not subjected to heat treatments or other sanitization during their preparation. The level of contamination at manufacturers is assured by the application of internal control procedures, adequate environmental hygiene, and personnel training. The spread of potential food spoilers or pathogens from environment to food is an ancient major concern in the food industry and recently, several studies have focused on the mapping of microbial contamination in food processing environments with the final aim to assess the types of microbes that can colonize the food processing environment and their abundance on surfaces and tools (Bokulich and Mills, 2013; Bokulich et al., 2013; Hultman et al., 2015; Stellato et al., 2015a,b; Calasso et al., 2016). Organic residues from food processing can create microenvironments for growth and accumulation of microorganisms that can be a relevant source of cross-contamination (Hood and Zottola, 1997; McLandsborough et al., 2006; Brooks and Flint, 2008).

*Pseudomonas* spp. are recognized as major food spoilers (Franzetti and Scarpellini, 2007; Ercolini et al., 2010; Martin et al., 2011; Doulgeraki et al., 2012; Marchand et al., 2012), they are psychrotrophic bacteria that easily develop in foods stored aerobically and at low temperatures such as meat, fish, milk, and dairy products (Nychas et al., 2008; De Jonghe et al., 2011; Remenant et al., 2015). *Pseudomonas* has been found as an abundant member of the microbiota in milk (De Jonghe et al., 2011; Marchand et al., 2012), beef (Ercolini et al., 2006, 2009), pork (Bruckner et al., 2012), chicken (Mellor et al., 2011), fish (Reynisson et al., 2008), and as a major contaminant of different surfaces (Bagge-Ravn et al., 2003; Brightwell et al., 2006; Licitra et al., 2007; Stellato et al., 2015b). Species such as *Pseudomonas fragi*, *P*. *fluorescens*, *P*. *putida, P. gessardii*, *P. lundensis* have been often isolated from spoiled foods and are currently recognized among the most threatening food spoilers (Ercolini et al., 2006, 2010; De Jonghe et al., 2011). Once they colonize the food matrix, they can be responsible for slime and malodour production that finally compromise food quality and consumer’s acceptability of the product (Nychas et al., 2008; Martin et al., 2011; Doulgeraki et al., 2012; Andreani et al., 2015; Casaburi et al., 2015; Pothakos et al., 2015). However, food spoiling potential is more than a species-specific trait.

Studies on fresh meat have demonstrated that diverse strains of the same species can behave differently in exactly the same food matrix and storage conditions. Accordingly, we have demonstrated that different biotypes can have different metabolic behaviors that will drive or not the spoilage-related activities and therefore determine whether the food spoilage will occur (Ercolini et al., 2010; Casaburi et al., 2011, 2014). Such biodiversity of *Pseudomonas* beyond the species level is surely reflected in colonization capability of the food processing environment, which is the major source of contamination. In fact, different *Pseudomonas* species and biotypes are characterized by resistance to routine cleaning of surfaces and tools and by capability for biofilm formation (Grobe et al., 2001; Wirtanen et al., 2001; Giaouris et al., 2014). Such traits make them ideal candidates to become resilient microbiota of the food processing environment.

The advances in technology and analytical tools for studies of microbial ecology have provided the possibility for in-depth studies of microbial diversity in food and food-related environments, and the characterization of microbial community structure in food science laboratories through 16S rRNA gene amplicon sequencing has been routinely applied (Ercolini, 2013). However, the taxonomic resolution of 16S rRNA gene-based surveys is generally limited to the genus level, and the common use of operational taxonomic units (OTUs) based on 97% sequence similarity cut-off often results in phylogenetically mixed units (Koeppel and Wu, 2013). These approaches in some cases fail to resolve ecologically meaningful differences between closely related organisms in complex environments (Eren et al., 2014, 2015). An alternative approach is oligotyping, which decomposes a given taxon, or 97% OTU, into high-resolution units (‘oligotypes’) by only using the most information-rich nucleotide positions identified by Shannon entropy calculations (Eren et al., 2013; Schmidt et al., 2014). Oligotyping has been applied to a wide range of ecosystems, including human (De Filippis et al., 2016) and animal guts (Menke et al., 2014), deep-sea sediments (Buttigieg and Ramette, 2015), and fresh water lakes (Newton and McLellan, 2015) to better understand the ecology of closely related microbial populations.

Given the ecological and health-related relevance of the biodiversity within *Pseudomonas* in food, here we used oligotyping to investigate the diversity of *Pseudomonas* populations in meat and dairy processing environments. Using a dataset of samples from the meat and dairy industry we investigated the *Pseudomonas* overlap between food and the food’s production environment and the existence of food- and environment-specific *Pseudomonas* types that can be possibly linked to resiliency in the food processing environment and to occurrence of food spoilage.

## Materials and Methods

### Sample Collection and Processing

A selection of samples from previous studies was used in order to assess the distribution of total *Pseudomonas* and individual *Pseudomonas* types in food and environmental samples. Two large datasets, one including samples from dairies (Stellato et al., 2015a; Calasso et al., 2016) and one with samples from meat processing plants (Ercolini et al., 2012; Stellato et al., 2016) were used, including both food and environmental specimens (Supplementary Table S1). After the genus-level taxonomic assignment, all the sample (see section “Oligotyping Analysis”) containing at least 0.08% of *Pseudomonas* were retained for this study. The dairy samples were collected from two different dairies and included cheeses and surface swabs (Stellato et al., 2015a; Calasso et al., 2016). Meat samples were collected from 20 butcheries (belonging to large and small retail) and included fresh beef and pork cuts and environmental swabs (Ercolini et al., 2012; Stellato et al., 2016). All sampling was performed in duplicate; after collection, samples were cooled at 4°C and analyzed within 3 h.

### Sequencing

The bacterial diversity was studied by pyrosequencing of the amplified V1–V3 region of the 16S rRNA gene, amplifying a fragment of 520 bp (Ercolini et al., 2012). Four hundred fifty four-adaptors were included in the forward primer followed by a 10 bp sample-specific Multiplex Identifier (MID). PCR conditions were previously described (De Filippis et al., 2013). After agarose gel electrophoresis, PCR products were purified twice by Agencourt AMPure kit (Beckman Coulter, Milano, Italy), quantified using the PlateReader AF2200 (Eppendorf, Milano, Italy) and equimolar pools were obtained prior to further processing. Amplicons were prepared and sequenced using a GS Junior platform (454 Life Sciences, Roche, Italy) according to the Roche standard protocols. The 16S rRNA gene sequences are available at the Sequence Read Archive of the NCBI (accession numbers SRP058584, SRP072347, SRP021108, SRP073300).

### Oligotyping Analysis

We used the oligotyping technique to explore differences within sequences identified for the genus *Pseudomonas*. For oligotyping analysis we used 308,842 quality-controlled V1–V3 reads from 197 samples. Raw reads were quality-filtered as follows: reads were trimmed at the first ambiguous base or when the average quality score dropped below 25 within a 50-bp-long window, and reads shorter than 500 bp and with >1 primer mismatch were discarded. The PyNAST algorithm (Caporaso et al., 2010) aligned the high-quality 454 reads against the GreenGenes (McDonald et al., 2012) multiple sequence alignment template (October 6, 2010 release) and alignment was further trimmed to equal length by using the o-smart-trim script included in the oligotyping package v. 1.0 (Eren et al., 2014). In order to avoid biases due to different sequencing depths, all samples were rarefied at 4500 reads after raw read quality filtering. Global Assignment of Sequence Taxonomy (GAST) algorithm (Huse et al., 2008) was used to identify “*Pseudomonas.*” Following the initial entropy analysis oligotyping was performed using version 2.1 of the oligotyping pipeline^1^ using a total of 14 positions with high entropy, chosen to compute the oligotypes (-C option). To minimize the impact of sequencing errors, we imposed that each oligotype must: (I) represent more than 1% of the reads for at least one sample, and (II) have at least 25 reads belonging to the most abundant unique sequence. After removal of oligotypes that did not meet these criteria, the analysis retained 299,055 reads (88.765% of the original reads). Oligotyping analysis identified 15 *Pseudomonas* oligotypes, representative sequences of which had at least one perfect match (100% sequence identity over 100% of query alignment) to rRNA gene entries in NCBI non-redundant (nr) database.

### Correlation Analyses

Correlation and co-occurrence tests within oligotypes were carried out using R (version 3.2.2) using the counts matrix for each oligotype (the number of reads assigned to each oligotype in each sample). Significance (*p* value) was calculated using the “corr” function, which employs a Student’s t distribution for a transformation of the correlation. We used the Bonferroni correction for multiple tests by multiplying significance estimates by 315^2^ ≅ 10^5^.

Two distinct indices (binary Jaccard: presence–absence and Morisita-Horn: relative abundance) estimated dissimilarity in pairwise comparisons of oligotype representative sequences.

Moreover, the pairwise sequence identity was calculated using o-sequence-distances and o-visualize-distance-matrix.R (from the oligotyping pipeline) to generate and visualize the distance matrix (i.e., percent identity matrix). The Morisita-Horn index was used for calculating the distance between each matrix cell (to get a “score” that represents row-wise similarity) and then clustered according to the Ward metric. The dendrogram represents the Morisita-Horn distances between each row/column, i.e., the distance between each pair of oligotype sequence. For all visualizations we used the ggplot2 package in R.

All the oligotypes were chosen for construction of a heatmap where samples and oligotypes were clustered based on Horn distances and Ward clustering.

## Results

### The Most Abundant *Pseudomonas* Oligotypes Are Shared between Meat and Cheese Datasets

**FIGURE 1:**
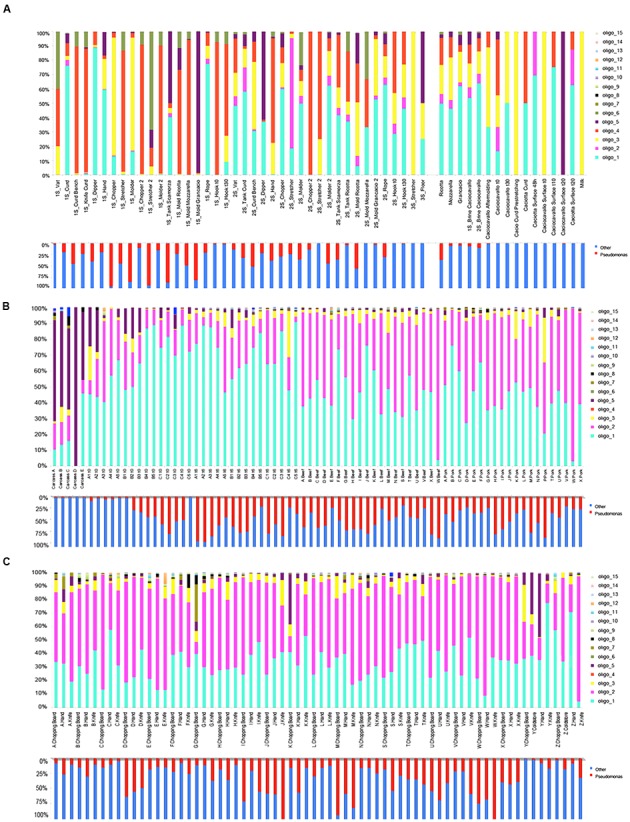
**Relative abundance of the *Pseudomonas* genus in the single samples (lower bars) and distribution of the corresponding oligotypes (upper). (A)** Cheese dataset; **(B)** Meat dataset, meat samples; **(C)** Meat dataset, environmental samples.

Two previous datasets including samples from dairies (Stellato et al., 2015a; Calasso et al., 2016) and meat processing plants (De Filippis et al., 2013; Stellato et al., 2016) were used in this study in order to map the distriution of *Pseudomonas* and its individual types in food and environmental samples. The two datasets included reads from the V1–V3 region of the 16S rRNA gene. Oligotyping analysis focused on *Pseudomonas* provided information that allowed taxonomic resolution beyond the genus level. We analyzed the distribution of each oligotype across the sampled environmental sites and among food products, and we characterized the oligotype composition of the manufactures within each processing plant. A total of 15 *Pseudomonas* oligotypes were identified across the different batches of sequences and they were associated into meat and cheese datasets. Comparing individual oligotype sequences with reference sequences in the NCBI’s nr database, we associated oligotypes to known species (**Table 1**). Many oligotypes distributed differentially across food and related environments, exhibiting a distinct community composition of *Pseudomonas* populations between cheese and meat datasets (Supplementary Figure S1). **Figure 1** displays the relative abundance of each *Pseudomonas* oligotype per sample with respect to the relative abundance of *Pseudomonas*. Abundance levels of the genus *Pseudomonas* are reported in the Supplementary Table S2. For the cheese dataset, *Pseudomonas* were more abundant in the environmental samples compared to cheese, while for the meat dataset *Pseudomonas* was particularly abundant in spoiled meat and in some specific tool surfaces, such as knife and chopping boards (**Figure 1**).

**Table 1:** *Pseudomonas* oligotypes identified by BLASTn search against the NCBI nr database.

Oligotype	Identification	Abundance in the cheese dataset (%)	Cheese (avg. %)	Environment (avg. %)	Abundance in the meat dataset (%)	Meat (avg. %)	Environment (avg. %)	Closest match accession number	Coverage (%)	Identity (%)
oligo_1	*P. fragi*	0.35–88.67	39.49	27.00	3.04–90.88	51.73	33.70	LK391512	100	100
oligo_2	*P. fragi*	0.28–76.95	6.34	4.13	3.09–96.19	35.30	54.41	CP013861	100	100
oligo_3	*P. fluorescens*	0.66–100	33.24	17.68	0.18–29.13	4.39	5.26	CP012400	100	100
oligo_4	*P. fluorescens*	0.52–100	12.33	34.51	0	0	0	AB968092	100	100
oligo_5	*P. jessenii*	0.44–100	8.24	9.59	0.01–100	7.05	3.42	LT547850	100	100
oligo_6	*P. fluorescens*	0.09–68.46	0.34	6.93	0	0	0	CP010945	100	100
oligo_7	*Pseudomonas* sp.	0	0	0	0.003–27.65	0.26	0.94	KT186220	100	100
oligo_8	*P. psychrophila*	0	0	0	0.01–10.17	0.29	0.68	JQ782900	100	98
oligo_9	*Pseudomonas* sp.	0	0	0	0.02–4.85	0.14	0.65	HQ189532	100	96
oligo_10	*P. fragi*	0	0	0	0.03–1.41	0.25	0.22	LK391512	100	98.6
oligo_11	*Pseudomonas* sp.	0	0	0	0.01–3.03	0.16	0.28	LC128308	100	94
oligo_12	*Pseudomonas* sp.	0	0	0	0.01–8.33	0.14	0.28	KT424974	100	96
oligo_13	*P. syringae*	0	0	0	0.02–5.77	0.21	0.09	KT783475	100	99
oligo_14	*P. fragi*	0	0	0	0.02–0.90	0.09	0.06	LK391512	100	99.7
oligo_15	*P. fluorescens*	0.07–1.28	0	0.14	0	0	0	CP005975	100	100


Interestingly, some oligotypes occurred both in cheese and meat samples and their related processing environments. The two datasets shared four oligotypes, although with differences in abundance. The five most abundant oligotypes (oligo_1–5) occurred in meat, cheese, and related environmental samples, except oligo_4, which was specific of the cheese dataset. Oligo_1 was highly abundant in both datasets, oligo_2 was more abundant in the meat, where the relative abundance of *Pseudomonas* genus was remarkable; on the contrary the oligo_3 showed higher levels in the cheese dataset although the relative abundance of the genus *Pseudomonas* was lower in this case. In addition, oligo_5 prevailed in carcasses, where the lowest incidence of *Pseudomonas* was found (**Figure 1**). Overall, it was evident that all the less abundant oligotypes were more specific for one or the other dataset; particularly oligo_6 and oligo_15 occurred only in the cheese and oligo_7–14 occurred only in the meat dataset, respectively (**Table 1** and **Figure 1**).

**FIGURE 2:**
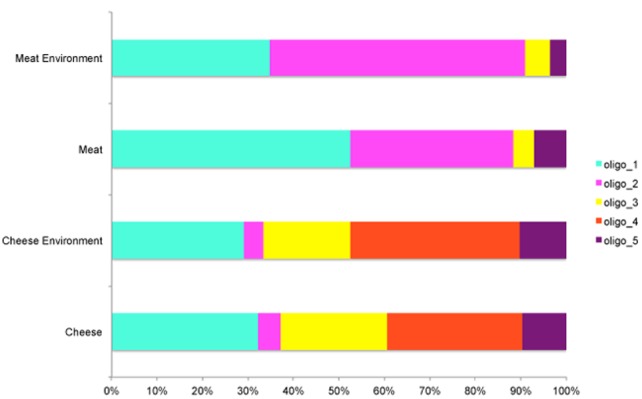
**Average levels of the most abundant oligotypes in the cheese and meat datasets**.

In both meat and cheese case and no oligotype was specific of food, they were all common to the corresponding processing environment (**Table 1**). The average levels of the most abundant oligotypes distributed across the meat and cheese dataset is represented in **Figure 2**. While in the cheese dataset there was no significant difference in oligotype abundance between cheese and cheese environment except for the oligo_4 (*p* < 0.005), in the meat dataset there was a significant increase of oligo_1 and a decrease of oligo_2 from environment to meat (*p* < 0.05; **Figure 2**).

### Oligotype Similarity and Correlations

**FIGURE 3:**
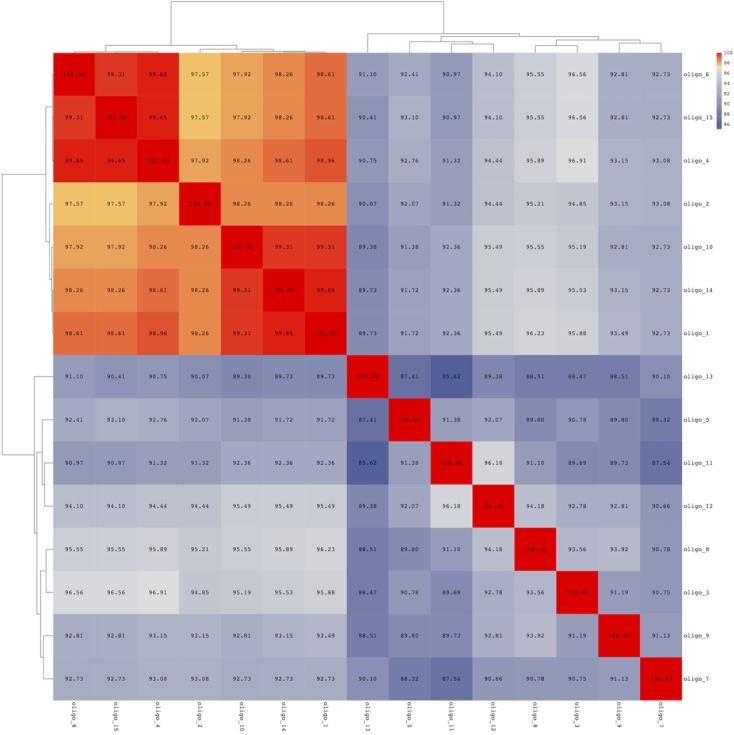
**Pairwise sequence identity for the oligotypes identified among cheese and meat samples.** Morisita-Horn was used for calculating the distance between each pairwise distance, clustering was obtained using the Ward metric.

Sequence distance matrix representation shows the percent nucleotide identity between each pair of oligotypes (**Figure 3**). The oligotypes are clearly divided into two groups, the first including all the most abundant and mainly those identified as either *P. fragi* or *P. fluorescens* and the second including less abundant oligotypes except oligo_3 (**Figure 3**). Sequence similarity within the first group were all >97%, confirming that the resolution allowed by oligotyping was necessary to distinguish these types. Correlation tests indicated that the most abundant oligotypes (oligo_1 and oligo_2) were strongly positively correlated in the cheese while they were negatively correlated in the meat dataset (Supplementary Figure S2). In addition, oligotypes 4 and 6 correlated positively (Supplementary Figure S2) with a major abundance in the environmental samples of the cheese dataset (**Figure 1**), while the abundance of oligo_1 was negatively correlated to that of oligo_4 that is typical of cheese dataset (Supplementary Figure S2). All the most abundant oligotypes in both food datasets (oligo_1–5) generally co-occurred; in particular, oligo_1 co-occurred with the other abundant oligotypes (oligo_2, 3, 4; Supplementary Figure S3).

## Discussion

Bacterial spoilage causes significant economic losses for the food industry and product contamination with psychrotrophic microorganisms is a particular concern for fresh foods. Dairy and meat products, beyond being among the most commonly consumed, can be also regarded as model foods to study the contamination routes of spoilage-associated bacteria. Psychrotrophic bacteria are ubiquitous in nature and can be isolated from soil, water, and vegetation (Dogan and Boor, 2003). Moreover, they can colonize meat and dairy products (Viljoen, 2001; Gram et al., 2002; Pennacchia et al., 2009) but generally constitute only a small amount of the initial microbiota in unprocessed food. Bacterial spoilage occurs when conditions during storage favor the growth of psychrotrophs and they become the dominant microbiota (Doulgeraki et al., 2012).

Not all *Pseudomonas* strains have the same abilities to produce defects in food (O’Sullivan and O’Gara, 1992; Dogan and Boor, 2003; Iulietto et al., 2015). Many spoilage-associated activities are strain-specific, and an accurate analysis of the processing environment is necessary in order to focus on those surfaces that may be source of specific spoilers (Brightwell et al., 2006; Licitra et al., 2007). Therefore, methods for discriminating strains with high food spoilage potential are necessary in order to identify and reduce or eliminate the environmental contamination sources of the critical strains. Although the 16S rRNA gene is a popular target to characterize bacterial community structures in environmental samples, high-throughput sequencing of short amplicons from this gene limits the taxonomic resolution that can be achieved. Here we used oligotyping (Eren et al., 2013) to decompose 16S tag sequences that were classified to the genus *Pseudomonas* into distinct, high-resolution oligotypes to investigate the diversity of this group in meat and dairy processing.

Although oligotyping is based on single nucleotide resolution and it covers the diversity captured in the sequenced region, it cannot address biological limitations of the 16S rRNA gene. It is likely that multiple distinct *Pseudomonas* strains occur, which are identical at the sequenced region of the 16S rRNA gene. Consequently, although an interesting level of resolution was achieved in this study, the *Pseudomonas* biodiversity may be still underestimated and some cosmopolitan and abundant oligotypes may represent aggregated strains of *Pseudomonas* with identical V1–V3 regions of the 16S rRNA gene. These limits might be overcome by using shotgun metagenomics and analyzing the whole genomes from the food and the corresponding environments, this would give a much higher discriminative power and allow to better characterize the *Pseudomonas* population or even to use a pangenomic approach for strain-level profiling.

We identified a total of 15 *Pseudomonas* oligotypes across dairy and meat samples as well as in related environmental samples from the processing plants. The four most abundant oligotypes were shared between the dairy and meat environment and showed high levels of correlation. Conversely, the least abundant were more food-specific. The ecological reason behind this result is relevant: the food environment selects specific *Pseudomonas* types that are capable of adaptation and only some can be actually found in food as spoilage agents. In both meat and dairy cases, *Pseudomonas* occurred both in the food matrices and in the processing environment. However, while in the case of cheese the oligotypes were more abundant in the environment, in the case of meat they showed higher levels in the meat. Among the cheeses, only the Ricotta cheese showed remarkable amounts of *Pseudomonas* while in all the others the relative abundance was negligible (**Figure 1**). Clearly, in spite of the high levels of *Pseudomonas* found in most of the environmental samples in the dairies, its occurrence in cheese is likely limited by the fermentation in cheese, which also explains why *Pseudomonas* is still abundant in Ricotta where no fermentation occurs. In addition, oligo_4 was only found in the dairy dataset, likely because of specific adaptation to this type of environment. Oligo_4 was identified as *P. fluorescens*, and strains of this species were previously associated to important cases of spoilage in dairy productions (Martin et al., 2011; Nogarol et al., 2013; Andreani et al., 2015). Episodes of cheese contamination from the environment can be therefore caused by selection of specific *Pseudomonas* types that become resilient in the processing environment and can contaminate the food causing spoilage. In the cheese dataset a higher diversity in oligotypes distribution across the samples was observed. The higher variability might be caused by the material used for the tools employed during cheese manufacturing (such as porous plastic gaskets); in fact, these materials may be more difficult to clean from organic residuals (Settanni et al., 2012; Montel et al., 2014).

On the other hand, the same *Pseudomonas* oligotypes increase in levels from environment to meat because in the meat matrix they find more favorable conditions to grow and become the dominant population. The most abundant oligotypes in meat belonged to *P. fragi*, very often associated to meat spoilage (Ercolini et al., 2007, 2010). Interestingly, *P. fragi* oligo_2 was much more abundant in the meat compared to the cheese dataset indicating specific adaptation capabilities to the food processing environments. In addition, two less abundant oligotypes in meat were identified as *P. fragi*, suggesting that different strains of the same species can have different adaptation efficiency and intra-species competition is likely to determine which strains will dominate in a specific environment. Oligo_5 identified as *P. jessenii* was the main oligotype in the carcasses but it was clearly outgrown by other types in both meat cuts and environment of production. This clearly indicates that independently from carcass microbiota at slaughter, the adapted *Pseudomonas* species and strains in the meat processing environment are the actual source of contamination, can actually become dominant in meat and, depending on storage conditions, determine spoilage.

The oligotype-level diversity and abundances were generally not correlated with the genus-level relative abundance of *Pseudomonas* in the samples. However, in cases such as those of the carcasses, low *Pseudomonas* abundance corresponded to dominance of a single oligotype indicating that competition between species of the same genus play a relevant role in the establishment of the dominant microbial population.

Species of the genus *Pseudomonas* are recognized as major food spoilers and exploring at sub-genus-level diversity can play a key role to investigate food contamination and development of spoilage since the capability to determine spoilage can be species- as well as strain-dependent. The approach followed here allowed us to investigate the previously unexplored diversity of the genus *Pseudomonas* in the food environment. Although the most frequent oligotypes are shared between the meat and dairy samples, their abundances are environment-specific, and the different oligotypes have defined co-abundance/exclusion patterns. We suggest this is a result of both food-specific adaptation and microbial competition leading to resilient bacterial associations that are likely involved in food contamination and spoilage.

## Author Contributions

GS performed experiments, analyzed the data and wrote the manuscript. DU analyzed the data and contributed to generation of tables and figures. AV analyzed the data and contributed to generation of tables and figures. MDA performed experiments and analyzed the data. AME contributed to study design, supervised data analysis, and wrote the manuscript. DE conceived and designed the study, analyzed the data and wrote the manuscript.

## Conflict of Interest Statement

The authors declare that the research was conducted in the absence of any commercial or financial relationships that could be construed as a potential conflict of interest.

## Supplementary Material

The Supplementary Material for this article can be found online at: http://journal.frontiersin.org/article/10.3389/fmicb.2017.00264/full#supplementary-material

## Footnotes

1 http://merenlab.org/projects/oligotyping/
