# Supplementary material for: A Few Pseudomonas Oligotypes Dominate in the Meat and Dairy Processing Environment
Source: Front Microbiol. 2017 Mar 2;8:264. doi: 10.3389/fmicb.2017.00264 (PMC5332365; doi:10.3389/fmicb.2017.00264)
Supplement: Supplementary file 1 [file Image_1.PDF]

Figure S1. Hierarchical average-linkage clustering of the samples based on the Pearson's correlation coefficient of the abundance of oligotypes in the different samples. The color scale indicates the scaled abundance of each variable: red, high abundance; blue, low abundance. The upper column bar is colored according to the type of sample: red, cheese dataset; blue, meat dataset.

Figure S2. Correlation between oligotypes occurring in the cheese (panel a) and meat (panel b) dataset. The bubbled text in each cell reports the p-value for that pairwise correlation. The color scale indicates the level of correlation (red, positive correlation; blue, negative correlation) and the cells are colored according to the correlation level. Tests were carried out using R (version 3.2.2) considering the counts matrix for each oligotype. Significance (*p* value) was calculated using the "corr" function, which employs a Student's t distribution for a transformation of the correlation. Bonferroni correction was used for multiple tests by multiplying significance estimates by $315^2 \sim= 10^5$.

Figure S3. Co-occurrence between *Pseudomonas* oligotypes occurring in the cheese (panel a) and meat (panel b) datasets. The bubbled text in each cell compares the number of samples in which both oligotypes co-occurred (numerator) to the prevalence of the more abundant oligotype (denominator). The color scale indicates the level of co-occurrence (purple; high number of co-occurrence; yellow, low number of co-occurrence) and the cells are colored according to the co-occurrence level. Tests were carried out using R (version 3.2.2) considering the counts matrix for each oligotype. Two distinct indices (binary Jaccard: presence–absence, and Morisita-Horn: relative abundance) estimated dissimilarity in pairwise comparisons of oligotypes sequences.

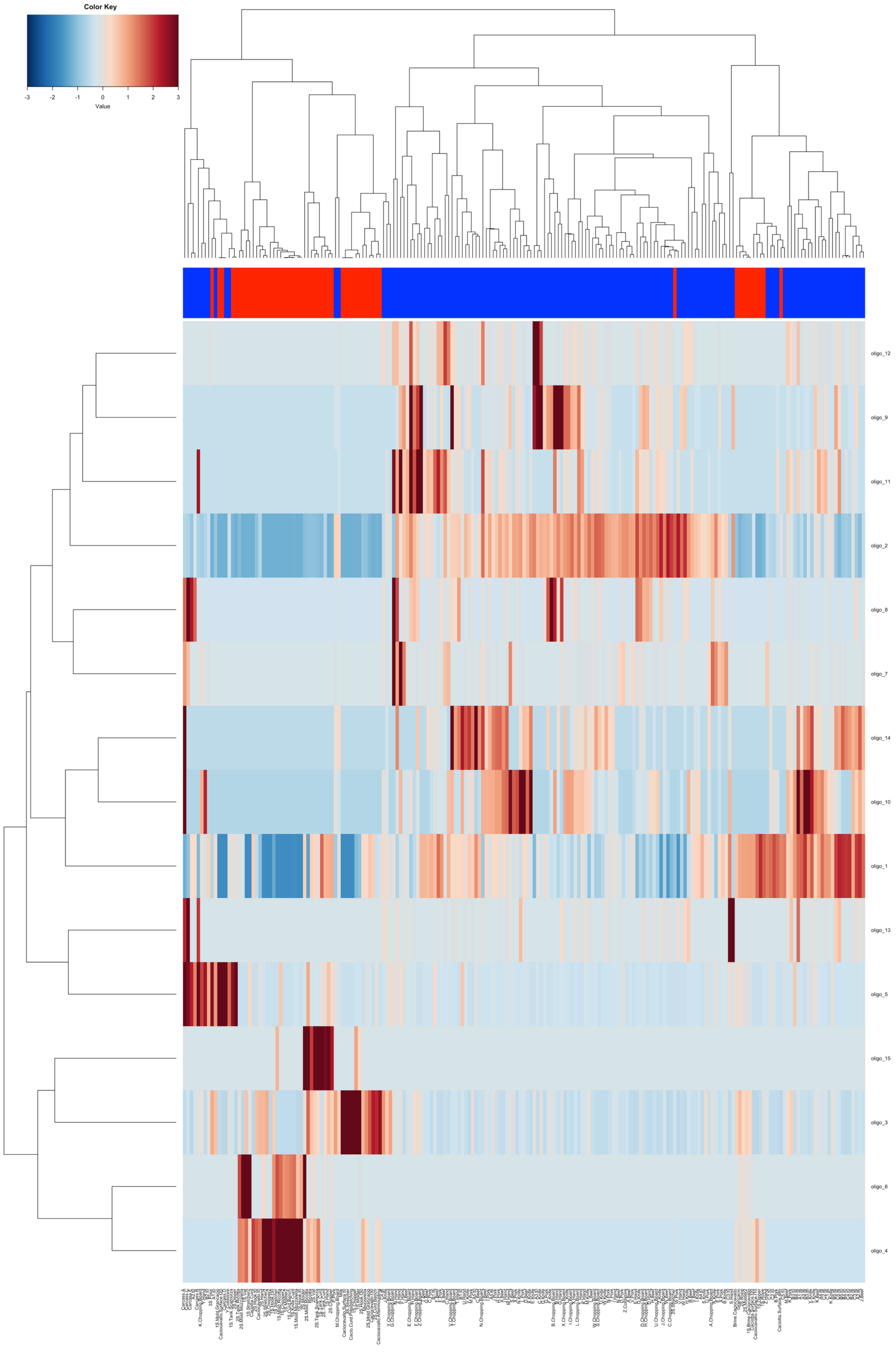
Color Key
-3
-2
-1
0
1
2
3
Value
oligo_12
oligo_9
oligo_11
oligo_2
oligo_8
oligo_7
oligo_14
oligo_10
oligo_1
oligo_13
oligo_5
oligo_15
oligo_3
oligo_6
oligo_4

Figure S1

Figure S2

a

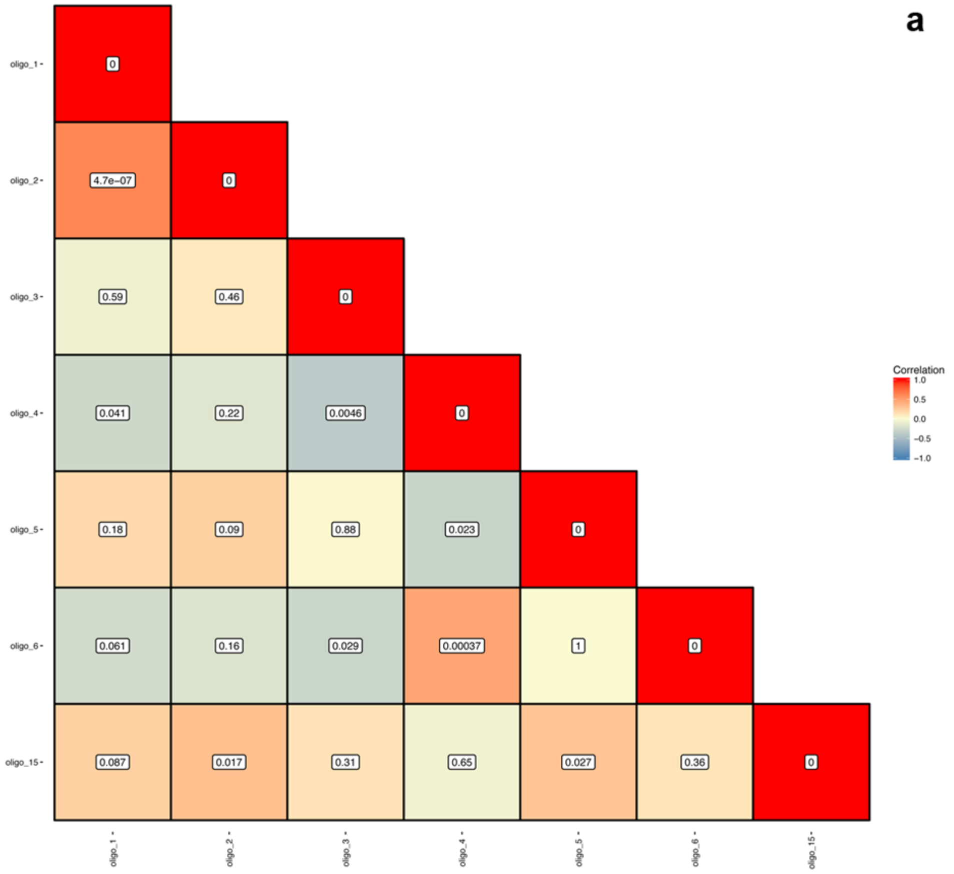

oligo_1
oligo_2
oligo_3
oligo_4
oligo_5
oligo_6
oligo_15
0
4.7e−07 0
0.59 0.46 0
0.041 0.22 0.0046 0
0.18 0.09 0.88 0.023 0
0.061 0.16 0.029 0.00037 1 0
0.087 0.017 0.31 0.65 0.027 0.36 0
Correlation
1.0
0.5
0.0
−0.5
−1.0

b

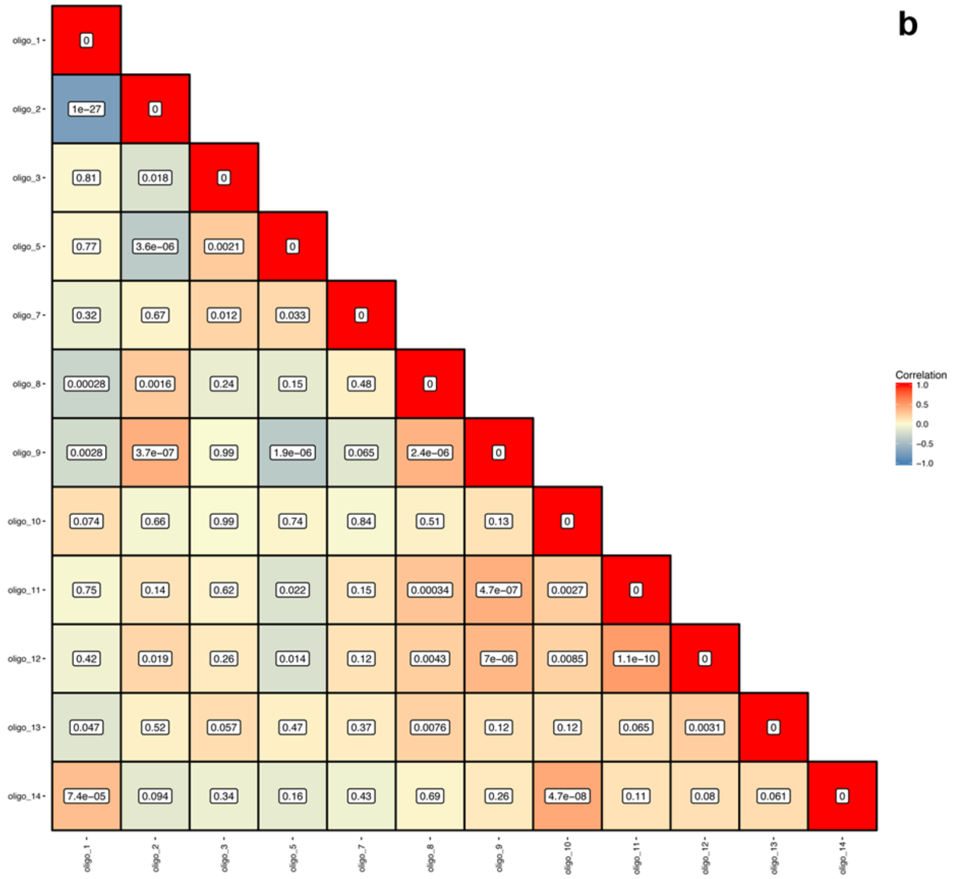

oligo_1
oligo_2
oligo_3
oligo_5
oligo_7
oligo_8
oligo_9
oligo_10
oligo_11
oligo_12
oligo_13
oligo_14
0
1e−27 0
0.81 0.018 0
0.77 3.6e−06 0.0021 0
0.32 0.67 0.012 0.033 0
0.00028 0.0016 0.24 0.15 0.48 0
0.0028 3.7e−07 0.99 1.9e−06 0.065 2.4e−06 0
0.074 0.66 0.99 0.74 0.84 0.51 0.13 0
0.75 0.14 0.62 0.022 0.15 0.00034 4.7e−07 0.0027 0
0.42 0.019 0.26 0.014 0.12 0.0043 7e−06 0.0085 1.1e−10 0
0.047 0.52 0.057 0.47 0.37 0.0076 0.12 0.12 0.065 0.0031 0
7.4e−05 0.094 0.34 0.16 0.43 0.69 0.26 4.7e−08 0.11 0.08 0.061 0
Correlation
1.0
0.5
0.0
−0.5
−1.0

Figure S3

a

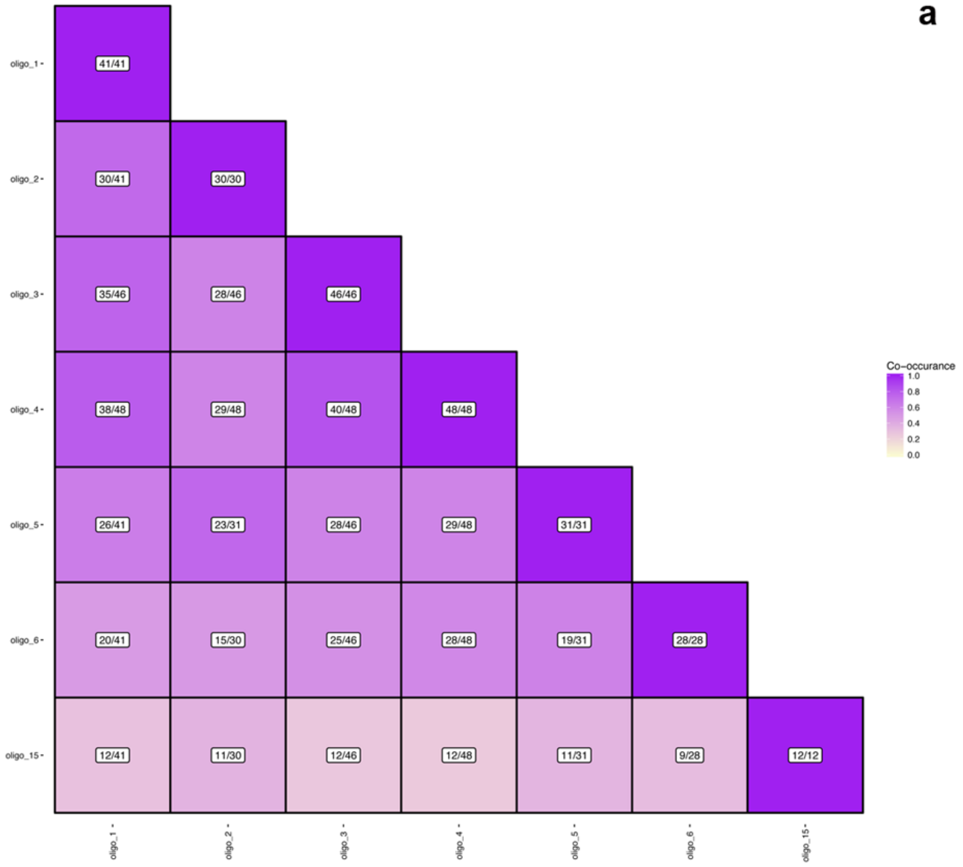
oligo_1 41/41
oligo_2 30/41 30/30
oligo_3 35/46 28/46 46/46
oligo_4 38/48 29/48 40/48 48/48
oligo_5 26/41 23/31 28/46 29/48 31/31
oligo_6 20/41 15/30 25/46 28/48 19/31 28/28
oligo_15 12/41 11/30 12/46 12/48 11/31 9/28 12/12
oligo_1 oligo_2 oligo_3 oligo_4 oligo_5 oligo_6 oligo_15
Co-occurance
1.0
0.8
0.6
0.4
0.2
0.0

b

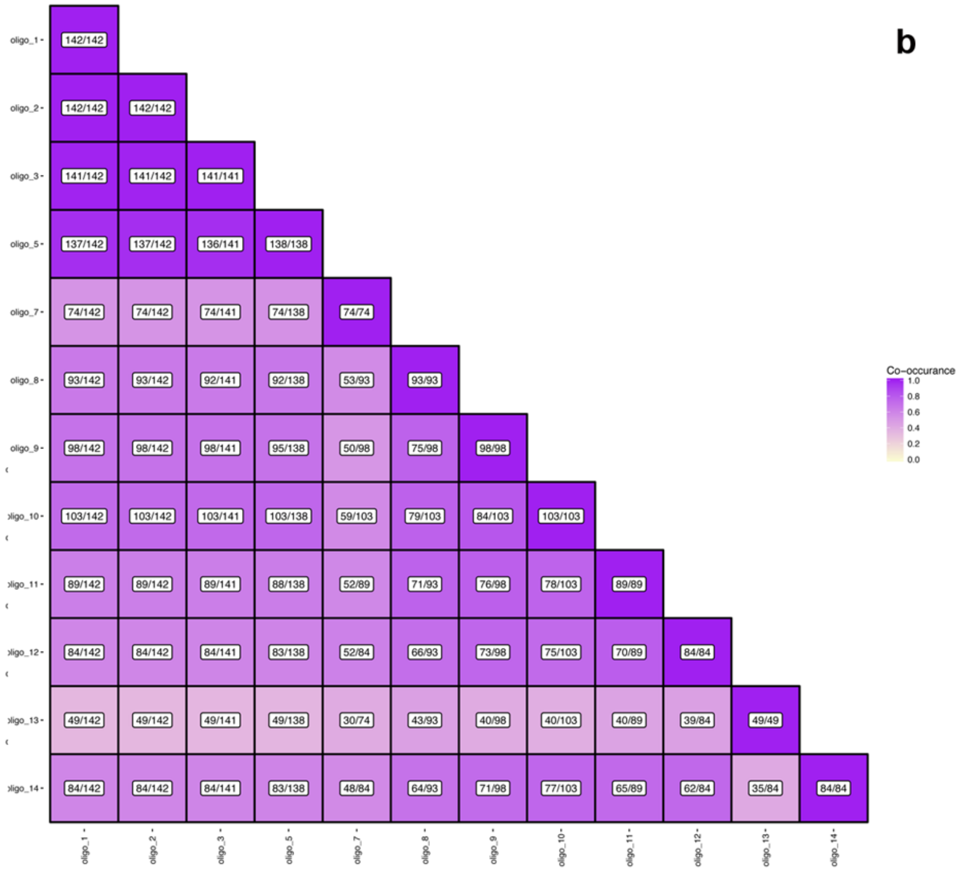
oligo_1 142/142
oligo_2 142/142 142/142
oligo_3 141/142 141/142 141/141
oligo_5 137/142 137/142 136/141 138/138
oligo_7 74/142 74/142 74/141 74/138 74/74
oligo_8 93/142 93/142 92/141 92/138 53/93 93/93
oligo_9 98/142 98/142 98/141 95/138 50/98 75/98 98/98
oligo_10 103/142 103/142 103/141 103/138 59/103 79/103 84/103 103/103
oligo_11 89/142 89/142 89/141 88/138 52/89 71/93 76/98 78/103 89/89
oligo_12 84/142 84/142 84/141 83/138 52/84 66/93 73/98 75/103 70/89 84/84
oligo_13 49/142 49/142 49/141 49/138 30/74 43/93 40/98 40/103 40/89 39/84 49/49
oligo_14 84/142 84/142 84/141 83/138 48/84 64/93 71/98 77/103 65/89 62/84 35/84 84/84
oligo_1 oligo_2 oligo_3 oligo_5 oligo_7 oligo_8 oligo_9 oligo_10 oligo_11 oligo_12 oligo_13 oligo_14
Co-occurance
1.0
0.8
0.6
0.4
0.2
0.0

Table S1 A - Description of the food samples analyzed in this study.

Sample	Description	Temperature (°C)	pH	a_w
[a]Brine Caciocavallo*	Liquid brine used for salting of Caciocavallo cheese	18	6.97	0.826
[b]Caciocavallo After moulding*	Caciocavallo cheese obtained after stretching and molding steps	65	5.64	0.976
[b]Caciocavallo Curd pre-stretching**	Curd obtained by coagulating milk and ready for stretching, molding and brining steps for Caciocavallo production	37	5.52	0.964
[b]Caciocavallo Surface t_0**	Section of Caciocavallo cheese after stretching, molding and brining, ready to start the maturation process	9	5.50	0.977
[b]Caciocavallo Surface t_{10}**	Section of Caciocavallo cheese after 10 days of ripening	9	5.79	0.951
[b]Caciocavallo Surface t_{20}**	Section of Caciocavallo cheese after 20 days of ripening	9	5.82	0.952
[b]Caciocavallo t_0 *	Caciocavallo cheese obtained after stretching, molding and brining, ready to start the maturation process	9	6.02	0.976
[b]Caciocavallo t_{30}*	Caciocavallo cheese after 30 days of ripening	9	5.60	0.954
[b]Caciotta Curd**	Curd obtained by coagulating milk addressed to Caciotta production	9	5.53	0.993
[b]Caciotta Surface 48h**	Shallow section of Caciotta cheese after 48 hours of ripening	9	5.51	0.979
[b]Caciotta Surface t_{20}**	Shallow section of Caciotta cheese after 20 days of ripening	9	5.17	0.941
[c]Milk*	Pasteurized milk addressed to cheese production	71	6.71	0.990
[b]Grancacio*	Pasta-filata cheese with production technology similar to Caciocavallo but characterized by larger size	9	5.63	0.916
[b]Mozzarella*	Mozzarella cheese	9	5.22	0.995
[b]Ricotta*	Fresh, soft cheese, not fermented	9	5.71	0.984
[b]Scamorza*	Pasta-filata cheese with a semi-soft texture and a typical pear shape	9	6.13	0.945
[d]Carcass (A-E)***	Swab sampling of bovine carcasses performed 12 hours from slaughtering, after washing/before chilling	7	6.81	0.995
[e]A 1-5.t_0***	Beef cuts from the butchery A were sampled immediately after portioning	4	5.44	9.983
[e]B 1-5. t_0***	Beef cuts from the butchery B were sampled immediately after portioning	4	5.72	0.985
[e]C 1-5. t_0***	Beef cuts from the butchery C were sampled immediately after portioning	4	5.32	0.986
[f]A 1-5.t_6***	Beef cuts from the butchery A were sampled after 6 days of storage from portioning	4	6.61	0.908
[f]B 1-5. t_6***	Beef cuts from the butchery B were sampled after 6 days of storage from portioning	4	7.11	0.912
[f]C 1-5. t_6***	Beef cuts from the butchery C were sampled after 6 days of storage from portioning	4	6.93	0.918
[e]A-W.Beef****	Beef cuts from the butchery were sampled immediately after portioning	4	5.24	0.986
[e]A-X.Pork****	Pork cuts from the butchery were sampled immediately after portioning	4	5.11	0.985

(A detailed description of the samples is reported by Stellato et al., 2015(*); Calasso et al., 2015(**);De Filippis et al., 2013(***); Stellato et al., 2016(****)).

Table S1 B - Description of the sampling method in this study.

Sample	Description
a	Fifty ml of liquid brine were sampled by sterile vessel
b	Representative section was sampled
c	Fifty ml of pasteurized milk were sampled by sterile vessel
d	The carcass was sampled rubbing with sterile sponge swab vertically, horizontally and diagonally across the sampling site (100 cm^2)
e	Fresh beef and pork cuts were sliced by knife and immediately analyzed once transferred to the laboratory
f	Beef and pork cuts were sliced by knife and immediately analyzed after 1 week of storage at 4°C

Tabella S2-A Relative abundance of *Pseudomonas* in the Cheese dataset samples.

Sample	*Pseudomonas* abundance (%)	Sample	*Pseudomonas* abundance (%)
1S_Vat	2.51	2S_Stretcher2	0.26
1S_Tank.Curd	15.82	2S_Molder2	64.01
1S_Curd.Bench	37.15	2S_Tank.Scamorza	28.75
1S_Knife.Curd	19.32	2S_Tank.Ricotta	3.36
1S_Dipper	32.53	2S_Mold.Ricotta	45.43
1S_Hand	16.29	2S_Mold.Mozzarella	0.15
1S_Chopper	77.98	2S_Mold.Grancacio	2.46
1S_Stretcher	36.06	2S_Rope	24.46
1S_Molder	31.07	2S_Hook.t0	0.11
1S_Chopper2	8.13	2S_Hook.t30	1.13
1S_Stretcher2	75.26	3S_Stretcher	0.11
1S_Molder2	12.24	3S_Floor	0.23
1S_Tank.Scamorza	70.53	Ricotta	29.88
1S_Mold.Ricotta	19.15	Mozzarella	4.84
1S_Mold.Mozzarella	39.96	Grancacio	4.13
1S_Mold.Grancacio	66.24	Brine.Caciocavallo	5.03
1S_Rope	16.58	1S_Brine.Caciocavallo	8.59
1S_Hook.t0	1.54	Caciocavallo.Aftermoulding	0.72
1S_Hook.t30	1.89	Caciocavallo.t0	0.32
2S_Vat	10.75	Caciocavallo.t30	0.09
2S_Tank.Curd	25.96	Cacio.Curd.Prestretching	0.05
2S_Curd.Bench	42.11	Caciotta.Curd	0.84
2S_Dipper	17.35	Caciotta.Surface.48h	0.11
2S_Hand	31.43	Caciocavallo.Surface.t0	0.14
2S_Chopper	23.91	Caciocavallo.Surface.t10	0.21
2S_Stretcher	19.55	Caciocavallo_Surface.t20	1.26
2S_Molder	29.02	Caciotta_Surface.t20	0.05
2S_Chopper2	11.36	Milk	0.49

Table S2-B Tabella S2-A Relative abundance of *Pseudomonas* in the meat samples.

Sample	*Pseudomonas* abundance (%)	Sample	*Pseudomonas* abundance (%)	Sample	*Pseudomonas* abundance (%)
Carcass.A	1.47	B1.t6	50.76	T.Beef	34.5
Carcass.B	1.64	B2.t6	61.15	U.Beef	36.77
Carcass.C	0.83	B3.t6	59.17	V.Beef	19.62
Carcass.D	0.21	B4.t6	33.44	X.Beef	70.76
Carcass.E	1.14	B5.t6	16.82	W.Beef	64.35
A1.t0	7.15	C1.t6	62.51	A.Pork	29.35
A2.t0	2.71	C2.t6	46.82	B.Pork	42.34
A3.t0	4.49	C3.t6	66.76	C.Pork	23.07
A4.t0	3.84	C4.t6	17.95	D.Pork	17.71
A5.t0	2.17	C5.t6	28.76	E.Pork	27.92
B1.t0	2.39	A.Beef	51.48	F.Pork	26.99
B2.t0	22.87	B.Beef	26.81	G.Pork	19.05
B3.t0	26.69	C.Beef	52.6	H.Pork	63.86
B4.t0	34.57	D.Beef	49.01	I.Pork	54.96
B5.t0	33.64	E.Beef	18.59	J.Pork	42.13
C1.t0	46.81	F.Beef	40.34	K.Pork	27.92
C2.t0	63.15	G.Beef	57.15	L.Pork	21.8
C3.t0	41.45	H.Beef	54.96	M.Pork	60.99
C4.t0	39.59	I.Beef	54.91	N.Pork	44.39
C5.t0	0.48	J.Beef	62.62	P.Pork	69.45
A1.t6	75.33	K.Beef	47.7	T.Pork	27.57
A2.t6	76.66	L.Beef	20.43	U.Pork	35.33
A3.t6	67.46	M.Beef	40.45	V.Pork	16.32
A4.t6	48.47	N.Beef	34.39	W.Pork	20.92
A5.t6	34.69	S.Beef	14.62	X.Pork	20.79

Table S2-C Relative abundance of *Pseudomonas* in the meat dataset: environmental samples.

Sample	*Pseudomonas* abundance (%)	Sample	*Pseudomonas* abundance (%)	Sample	*Pseudomonas* abundance (%)
A.Chopping.Board	6,26	H.Knife	8,45	T.Hand	27,34
A.Hand	20,97	I.Chopping.Board	56,23	T.Knife	34,21
A.Knife	8,16	I.Hand	16,24	U.Chopping.Board	42,84
B.Chopping.Board	11,84	I.Knife	43,51	U.Hand	54,9
B.Hand	23,87	J.Chopping.Board	46,07	U.Knife	32,22
B.Knife	8,16	J.Hand	47,17	V.Chopping.Board	16,86
C.Chopping.Board	11,84	J.Knife	80,93	V.Hand	16,72
C.Hand	8,11	K.Chopping.Board	12,32	V.Knife	46,68
C.Knife	3,43	K.Hand	44,86	W.Chopping.Board	59,14
D.Chopping.Board	50,23	K.Knife	12,29	W.Hand	53,68
D.Hand	43,54	L.Chopping.Board	23,58	W.Knife	84,79
D.Knife	38,85	L.Hand	12,24	X.Chopping.Board	31,3
E.Chopping.Board	14,31	L.Knife	9,43	X.Hand	34,17
E.Hand	11,06	M.Chopping.Board	74,41	X.Knife	30,75
E.Knife	11,54	M.Hand	46,49	Y.Chopping.Board	0,55
F.Chopping.Board	17,46	M.Knife	65,03	Y.Coldstore	1,94
F.Hand	5,92	N.Chopping.Board	12,6	Y.Hand	6,16
F.Knife	20,28	N.Hand	12,24	Y.Knife	13,44
G.Chopping.Board	8,67	N.Knife	19,55	Z.Chopping.Board	2,45
G.Hand	8,84	S.Chopping.Board	14,33	Z.Coldstore	14,07
G.Knife	29,34	S.Hand	42,53	Z.Hand	6,61
H.Chopping.Board	14,34	S.Knife	22,45	Z.Knife	25,36
H.Hand	28,22	T.Chopping.Board	27,33		
